# Novel Small Molecule Inhibitors Targeting the IL-6/STAT3 Pathway or IL-1β

**DOI:** 10.3390/molecules27092696

**Published:** 2022-04-22

**Authors:** Jihye Yoo, Darong Kim, Jiyoung Park, Young-Kook Kim, Hea-Young Park Choo, Hyun Ae Woo

**Affiliations:** 1 Graduate School of Pharmaceutical Sciences, College of Pharmacy, Ewha Womans University, Seoul 03760, Korea; jibong9077@ewhain.net (J.Y.); kdrlj237@ewhain.net (D.K.); jypark89@ewhain.net (J.P.); 2 Fluorescence Core Imaging Center, Department of Life Science, Ewha Womans University, Seoul 03760, Korea; 3 Laboratory Animal Resource Center, Korea Research Institute of Bioscience & Biotechnology (KRIBB), Daejeon 34141, Korea; kimyk@kribb.re.kr

**Keywords:** 2,5-diaminnobenzoxazole, anti-inflammatory effect, rheumatoid arthritis, IL-6, IL-1β, zymosan A, small molecule inhibitors

## Abstract

Development of small molecules that inhibit inflammatory cytokines is a desirable strategy for the treatment of inflammatory diseases such as rheumatoid arthritis (RA). Following up a previous study, we synthesized 10 novel compounds with a 2,5-diaminobenzoxazole moiety and evaluated their biological activities. Among them, compound 3e showed potent inhibitory activity on Interleukin 6 (IL-6)/Signal Transducer and Activator of Transcription 3 (STAT3) signaling inhibition (71.5%), and 3a showed excellent inhibitory activity on Interleukin 1 (IL-1β) (92.1%). To test in vivo anti-inflammatory activity, compounds 3a and 3e were administered by intraperitoneal (IP) injection after subcutaneous (SC) injection of zymosan A into the right footpad of mice. Inflammation on the footpad was reduced after administration of compounds 3a and 3e. Especially, compound 3a showed a significant ameliorative effect on zymosan-induced inflammation. From the in vivo and in vitro test results, we confirmed that our synthesized compounds are effective on the RA animal model through inhibition of the IL-6/STAT3 signaling pathway. Since drugs developed with small molecule inhibitors have several advantages over biological drugs, further study on these compounds is needed for the development of potent SMI drugs on RA.

## 1. Introduction

Interleukin-6 (IL-6) is a pleiotropic cytokine that has significant functions in regulation of the immune system [1]. It is a 21–28 kDa glycoprotein consisting of 185 amino acids. IL-6 is mostly generated from macrophages, fibroblasts, vascular endothelial cells, and other various cells such as T cells, B cells, cartilage cells and osteoblasts [2,3]. IL-6 plays both a proinflammatory and anti-inflammatory role in humans [4]. IL-6 is also important in the transition between acute and chronic inflammation [5].

IL-6 is involved in intracellular signal transduction in two ways. First, classical IL-6 receptor signaling is a method of signal transduction via membrane-bound IL-6 receptor (IL-6R) subunit. Second, IL-6 trans-signaling is a process in which a soluble form of IL-6R (sIL-6R) binds secreted IL-6 to make a complex that augments the half-life of IL-6 and enhance its bioavailability. IL-6 plays an important role in anti-inflammatory and repair processes via classical signaling under normal conditions, while the trans-signaling is involved in the pathogenesis of inflammatory disease [6]. The intracytoplasmic portion of IL-6R does not have tyrosine kinase domains and an enzymatic role in IL-6 signaling. Instead, when IL-6 binds to the receptor, 130 kDa glycoprotein (gp130) in the cell binds to IL-6R, enabling intracellular signaling. In this process, a multimeric complex of two IL-6 molecules, two IL-6R molecules and two gp130 molecules is formed, and dimerized gp130 induces tyrosine phosphorylation of STAT3 by signaling intracytoplasmic JAK tyrosine kinases (Janus family tyrosine kinases). Phosphorylated STAT3 translocates to the nucleus and induces gene expression [7,8,9].

In rheumatoid arthritis (RA) pathogenesis, the cytokine network is complicated, with various cytokines in blood and synovial joints. Among these cytokines, RA is characterized by systemic inflammation with elevated levels of serum IL-6 [10,11]. IL-6 promotes synovitis and joint destruction by stimulating neutrophil migration and osteoclast maturation. IL-6/sIL-6r complex also increases bone resorption, which further leads to pannus formation. Injured joints promote inflammation and formation of ectopic lymphoid follicles that produce autoantibodies and exacerbate synovitis [12].

IL-6 plays a significant role in B-cell maturation and production of auto-antibodies, as well as the stimulation of C-reactive protein (CRP) from hepatocytes. In an animal model of autoimmune diseases, IL-6 acts as a modulator of Th17 pro-inflammatory lymphocytes. In patients with RA symptoms, synovial fluid and synovium contains an elevated level of IL-6 compared to control patients with osteoarthritis. Further, increased activity of IL-6 correlates with elevations of acute-phase reactants in inflammation, such as fever and anemia.

IL-6 has been known to be involved in acquired immunity and there have been many studies on the pathogenic role of IL-6 in arthritis. IL-6 plays an important role in the differentiation of B lymphocytes into auto-antibody producing plasma cells, which are involved in the pathogenesis of arthritis by immune complex (IC) formation [13]. Further, high concentrations of IL-6 in RA may lead to Th17/Treg cell imbalance.

IL-6 mediated dysregulation of B lymphocytes and T lymphocytes may result in the abnormal secretion of inflammatory cytokines, chemokines, and metalloproteases from both lymphocytes and synovial stromal cells, resulting in synovial tissue degradation and bone erosion. Recently, however, studies have shown that IL-6 amplifies the toll-like receptor (TLR)-induced inflammatory response in RA pathogenesis [14]. TLRs play a key role in the innate immune system and bind to various kinds of bacterial and viral peptides. Signals from these TLR ligands generally activate mitogen-activated protein kinases (MAPKs), p65 nuclear-factor kappa B (p65 NF-kB), and interferon regulatory factor 3 (IRF-3). Inflammatory cytokines and type I interferon secretion are promoted by these processes [15]. For this reason, blocking the IL-6 signaling pathway may be an essential strategy in terms of both innate and acquired immunity for treating inflammatory diseases such as RA.

IL-6-involved inflammation can be inhibited by decreasing the synthesis of IL-6 or by blocking IL-6-mediated signal transduction. Inhibition of IL-6 through monoclonal antibodies is useful in the treatment of RA. Tocilizumab and Siltuximab are monoclonal antibodies used as drugs to ameliorate the symptoms of RA [16,17]. However, because of the high cost of monoclonal antibodies, the search for small molecular IL-6 inhibitors is needed. Several synthetic and natural small molecules, including madindolin A, are reported as IL-6 inhibitors. Tofacitinib is the only small molecular drug approved for treatment of RA. It is a JAK inhibitor that interferes with the JAK-STAT signaling pathway, which transmits extracellular information into the cell nucleus, influencing DNA transcription.

Previously, we reported the design and synthesis of a novel series of 2,5-diaminobenzoxazole derivatives which have suppressive activity on IL-6/STAT3 signaling [18]. Among the compounds, *N*-(4-ethylphenyl)benzo[*d*]oxazole-2,5-diamine (compound 4 in the literature) showed a significant inhibition effect of 97.5% (at 20 µg/mL). The compound was also effective against other inflammatory cytokines such as IFN-γ, IL-2, IL-17, IL-4, interleukin-5 (IL-5) and interleukin-13 (IL-13) which was generated from T helper cells (Th1, Th17 and Th2 cells). In this study, we prepared 2,5-aminobenzoxazole derivatives with smaller substituents at the fifth amino group by referring to the result of the previous study. We investigated whether the IL-6/STAT3 pathway was inhibited by the synthesized compounds, and then we examined the effect of zymosan-induced arthritis in a rat model.

In addition, various cytokines such as interleukin-1β (IL-1β), tumor necrosis factor-α (TNF-α), are produced during inflammatory processes that act in synergy and have overlapping activity with IL-6. We also examined the suppressive effect of the compounds on IL-1β and IL-6 production on macrophages.

## 2. Results

### 2.1. Synthesis of 2,5-Diaminobenzoxazole Derivatives

**Scheme 1:**
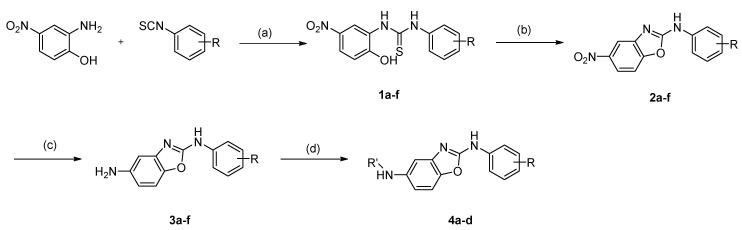
Synthesis of 2,5-diaminobenzoxazole derivatives. (**a**) MeOH, rt, 24 h, (**b**) KO_2_, acetonitrile, N_2_, rt, 16 h, (**c**) SnCl_2_, ultrasonication, rt, 3 h, (**d**) Acid chlorides or formaldehyde/sodium triacetoxyborohydride.

Novel compounds with 2,5-diaminobenzoxazole moiety were synthesized as shown in Scheme 1. Commercially available 2-amino-4-nitrophenol was reacted with variously substituted phenyl isothiocyanates to produce thiourea compounds (1a–f). The thioureas were cyclized with KO_2_ to obtain benzoxazoles by oxidation (2a–f). The nitro group was reduced to the amino group with tin (II) chloride (3a–f). The amino group was then reacted with various acid chlorides, acid anhydride or formaldehyde to afford the desired amides (4a–d).

### 2.2. Inhibition of IL-6/STAT3 Signaling and Production of IL-1β by Benzoxazole Derivatives

We screened 10 novel synthetic compounds (compounds 3a–f and 4a–d) by measuring the effects of each compound on IL-6-induced luciferase expression in human hepatocarcinoma HepG2 cells transfected with *p*-STAT3-Luc. In brief, *p*-STAT3-Luc-transfected HepG2 cells were stimulated with IL-6 in the presence of screening compounds, and luciferase activities were measured. The 2,5-diaminobenzoxazole derivatives showed 9.6–71.5% inhibition against IL-6/STAT3. The results are shown in Table 1. Among them, *p*-butyl substituted compound 3e showed the best inhibition activity at the concentration of 10 μg/mL (71.5%). The *p*-methoxy substituted compound 3a showed 29% inhibition on IL-6, but 92.1% inhibition on Il-1 β. Madindolin A, a known selective inhibitor of IL-6, showed 52% inhibition at 10 μg/mL. These compounds, 3e and 3a, exhibited the IC_50_ values of 3.51 μg/mL and 8.99 μg/mL, respectively. The IC_50_ value of Madindolin A was 8.70 μg/mL. We also examined the suppressive effect of the compounds on IL-1β production on macrophages. (Table 1) The compounds 3a, 4a, and 4c showed a significant inhibitory effect (92.1%, 88.2%, and 88.1%, respectively) on the production of IL-1β from macrophages at 10 μg/mL.

Based on the results in Table 1, we performed in vitro tests using 3a or 3e. The compound 3e showed a significant inhibition effect in inhibiting the secretion of both IL-6 and IL-1β from macrophages, and compound 3a showed a potent inhibition effect about IL-1β (Table 1). To assess the inhibitory effects of compounds on the LPS-induced secretion of IL-6 or IL-1β mRNA in Raw 264.7 cells, we tested their dose-dependent inhibitory effects on macrophage cell viability. Neither substance showed toxicity at concentrations below 100 μg/mL (data not shown). Thus, we examined their inhibition effect on IL-1 beta or IL-6 mRNA levels caused by LPS in two types of cells using a concentration range, where no cytotoxicity was observed. Both of them decreased the LPS-induced mRNA levels of IL-1 beta or IL-6 in cells in a dose-dependent manner (Table 2). Next, we examined whether two types of compounds induced inhibition effect of IL-1 beta or IL-6 more efficiently in combination compounds than either compound does alone in cell lines (Table 2). Compounds in combination induced no synergistic inhibition effect of IL-1 beta or IL-6 on cells.

### 2.3. Anti-Inflammatory Activity of Compounds 3a and 3e in an Animal Model

**Figure 1:**
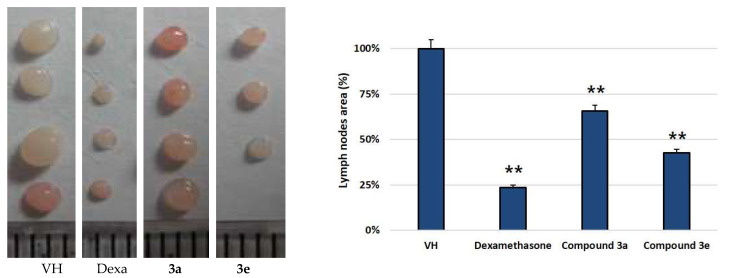
Zymosan A-induced infiltration of inflammatory cells into draining pLNs (VH: control group injected zymosan A only) and the effect on pLN reduction by the compounds. The anti-inflammatory drug dexamethasone was employed as a positive control. Samples were administered by IP from day 0 to day 6. On day 7, pLNs were isolated and weighed. ** *p* < 0.01 compared with the group treated with the LPS alone. Data are presented as the mean ± SD.

**Figure 2:**
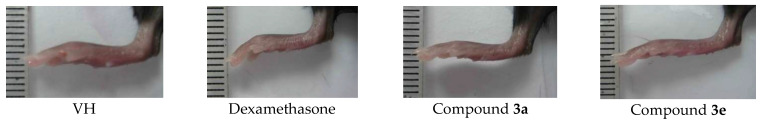
Shape of the mouse foot. Zymosan A-induced infiltration of inflammatory cells and edema was observed after the injection of zymosan A (VH) on the right footpad.

For the in vivo assay of anti-inflammatory activity in mice, two compounds (3a and 3e) were selected based on IL-6 and IL-1β assay data and were evaluated for their effect on draining lymph nodes and on footpad thickness. Zymosan A (3 mg/mL) was injected into the right footpad of the mice [19]. Zymosan A-induced infiltration of inflammatory cells into draining popliteal lymph nodes (pLNs) and caused their mass to increase. Further, edema was observed after the injection into the right footpad. The edema and sizes of pLNs were decreased with the treatment of compounds 3a and 3e (30 mg/kg), as shown in Figure 1 and Figure 2. Compound 3e showed a significant ameliorative effect, which is comparable to dexamethasone treatment. The anti-inflammatory drug dexamethasone was employed as a positive control.

**Figure 3:**
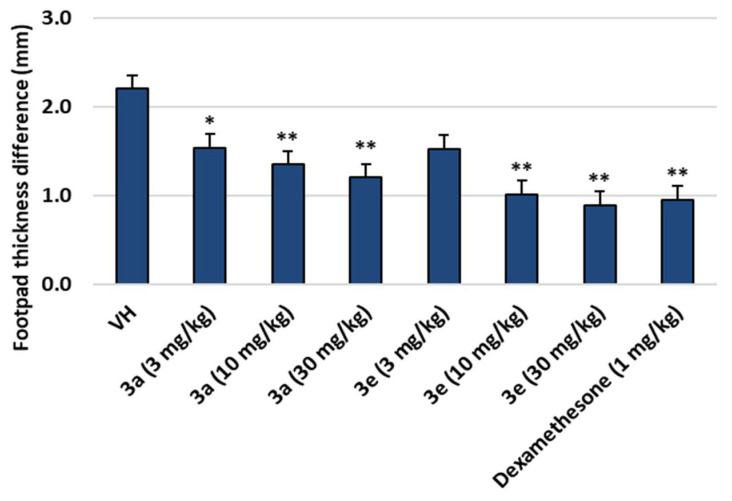
Footpad thickness differences in mice after treatment with compounds 3a and 3e (VH: zymosan A only). Data are presented as the mean ± SD. Student’s t-test was used for comparisons involving two groups. (*n* = 8). * *p* < 0.05, ** *p* < 0.01 vs. VH.

Footpad thickness of mice after treatment of compounds 3a and 3e decreased dose-dependently as shown in Figure 3. When the footpad thickness of untreated left foot and treated right foot was measured, the difference was 0.9~1.5 mm for 3e and 1.2~1.5 mm for 3a. There were no weight changes and outward toxicological symptoms of the mice with the treatment of the compounds.

## 3. Discussion

In this study, we synthesized 10 novel compounds with 2,5-diaminobenzoxazole moiety. The inhibition activity of the prepared compounds on the IL-6/STAT3 pathway showed various results ranging from 13–71% at 10 μg/mL concentration. Compound 3e, with no substituent on the amino group of the fifth position and an *n*-butyl group of *para*-position on benzene ring showed the best inhibition activity.

Since cytokines such as IL-1β, TNF-α, and nitric oxide (NO) are produced during inflammatory processes that act in synergy and have the overlapping activity with IL-6, we confirmed through additional in vitro test. Compound 3e showed a significant inhibition effect in inhibiting the secretion of both IL-6 and IL-1β from macrophages, and compound 3a showed a potent inhibition effect on IL-1β. However, the prepared compounds did not inhibit TNF-α production or NO production (data not shown).

We conducted an anti-inflammatory activity test on mice with compounds 3a and 3e. To understand RA pathogenesis and set a novel treatment strategy, several experimental models were developed that mimic arthritic process induced by collagen, zymosan, or antigen [20]. Compounds 3a and 3e were administered at three concentration levels (3, 10, 30 mg/kg) to the mice by IP injection. After 7 days of administration, the increment of pLN weight (ΔpLN weight) decreased upon the elevation of concentration. In particular, when treated with the compounds at 30 mg/kg, the anti-inflammatory effect was comparable to that of dexamethasone (1 mg/kg). Since the body weight was not changed during the test, the compounds only affected the inflammation region, not the entire body. The thickness of the footpad of the experimental groups (compound 3a and 3e administration) was comparable to those of control group. At the end of the test, we sacrificed the mice and compared the size of popliteal nodes (pLNs). The pLNs of the experimental groups were swollen compared to those of control group. However, they were smaller than that of the zymosan A only-administered group.

The IL-6 signaling pathway plays a key role in inflammation, and compounds inhibiting IL-6 signaling can be effective at preventing and treatment of chronic inflammatory diseases. IL-6 exerts its pleiotropic activity by activation of gp130 via binding to the transmembrane or soluble IL-6 receptor. IL-6 ligation of its receptor permits the intracellular segment to associate with gp130 in the form of a multimeric complex comprised of two IL-6 molecules, two IL-6R proteins and two gp130 proteins. Dimerized gp130 transduces a signal to activate intracytoplasmic JAK tyrosine kinases, which recruit and induce tyrosine phosphorylation of STAT3. Translocation of STAT3 to the nucleus induces new gene expression [21,22,23]. This JAK/STAT signaling has been demonstrated to regulate many kinds of diverse cellular functions critical to RA pathogenesis and progression, including cell survival and proliferation, determination of immune cell fate and apoptosis [24,25]. In normal conditions, JAK/STAT signaling is influenced by its negative regulators such as the suppressor of cytokine signaling (SOCS) and protein inhibitor of activated STAT. However, in RA, these regulators are abnormal. Continuous activation of JAK/STAT signaling in RA synovial joints results in an increased level of matrix metalloproteinase gene expression, increased frequency of apoptotic chondrocytes and apoptosis resistance in the inflamed synovial tissue [8].

## 4. Conclusions

There have been many studies to develop small molecule inhibitors for treatment of RA by regulation of the JAK/STAT signaling pathway [26]. The development of small molecule inhibitors of the JAK/STAT signaling pathways has recently been the target of additional pre-clinical experimental arthritis studies and RA clinical trials assessment. Tofacitinib, a JAK small molecule inhibitor, is now generally used to treat moderate to severe RA patients who have not adequately responded to disease-modifying anti-rheumatic drugs or various biologic agents [27,28].

The Benzoxazole moiety has oxygen and nitrogen atoms as hydrogen acceptors in its scaffold that exhibit numerous noncovalent interactions with protein targets. It also has a planar geometry that results in the π-π stacking, π-cation, and hydrophobic interactions with biomolecules. Especially, benzoxazoles with substituents at the 2-position have been found to have a remarkable impact on biological activities. A recent study showed that some enzoxazole derivatives act as TLR9 inhibitors with moderate to excellent activity in short term suppression of IL-6, suppression of IL-6 release from lymph nodes, indicating their potential as therapeutic agents for rheumatoid arthritis [29].

In this study, we also found that the prepared compounds inhibit the production of IL-1β, since activated macrophages are the key effector cells in RA pathogenesis, and these activated macrophages secrete proinflammatory cytokines such as IL-6 and IL-1β and give rise to localized and systemic inflammation [30]. For this reason, many kinds of anti-rheumatic drugs that block IL-1β or target IL-1β receptors have been studied, but no small molecule inhibitors have been introduced on the market [31,32,33]. Recently, IL-1 β has been found to be involved in periodontal pathogenesis. Therefore, inhibiting the production of IL-1β is expected to be effective in the treatment of periodontitis [34].

From the in vivo and in vitro test results, we confirmed that our synthesized compounds are effective in the RA animal model by inhibition of the IL-6/STAT3 signaling pathway and/or inhibition of IL-1β production. Even though further mechanistic study on gp130, JAK 1/2/3, and STAT3 phosphorylation is needed, it is possible to develop small molecule inhibitors from the prepared compounds.

## 5. Materials and Methods

### 5.1. Materials and Methods

Melting points were measured on an electro thermal digital melting point (Büchi, Germany) without calibration. ^1^H-NMR spectra were recorded on Varian NMR AS and Varian Unity Inova 400 MHz NMR spectrometers. Chemical shifts were reported in parts per million (*δ*) units relative to the solvent peak. The ^1^H NMR data were reported as peak multiplicities: s for singlet; d for doublet; t for triplet; q for quartet and m for multiplet. Coupling constants were recorded in Hertz (Hz). MS spectra were measured using a Jeol JMS 700 high resolution mass spectrometer from the Korea Basic Science Institute (Daegu). Reagents were of commercial grade and were purchased from Sigma-Aldrich Co., Merck, (St. Louis, MO, USA) and Duksan Pure Chemical Co., (Seoul, Korea).

### 5.2. Synthesis of 1-(Substituted phenyl)-3-(2-hydroxy-5-nitrophenyl)thioureas (1a–f)

Methanol (25 mL) was added to 2-amino-4-nitrophenol (100 mg, 1 eq.) and variously substituted isothiocyanates (1 eq.). The reaction mixture was stirred at room temperature for 24 h. After reaction, the organic solvent was removed under reduced pressure to obtain 1-(substitutedphenyl)-3-(2-hydroxy-5-nitrophenyl)thioureas (1a–f).

1-(2-Hydroxy-5-nitrophenyl)-3-(4-methoxyphenyl)thiourea (1a)

Yellow solid (75%), m.p. 127~132 °C, ^1^H-NMR (DMSO-*d*_6_, 400 MHz) *δ* 11.66 (s, 1H), 10.14 (s, 1H), 9.25 (d, *J* = 2.8 Hz, 1H), 9.16 (s, 1H), 7.92 (dd, *J* = 9.2, 2.8 Hz, 1H), 7.39 (dd, *J* = 6.8, 2.0 Hz, 2H), 7.02 (d, *J* = 8.8 Hz, 1H), 7.94 (dd, *J* = 6.8, 2.4 Hz, 2H), 3.76 (s, 3H), HR-FABMS Calcd for C_14_H_14_N_3_O_4_S (M^+^ + H): 320.07, Found: 320.07.

1-(4-Ethoxyphenyl)-3-(2-hydroxy-5-nitrophenyl)thiourea (1b)

Light yellow solid (19%), m.p. 155~157 °C, ^1^H-NMR (Acetone-*d*_6_, 400 MHz) *δ* 10.09 (s, 1H), 9.33 (d, *J* = 15.2 Hz, 1H), 8.63 (s, 1H), 7.96 (dd, *J* = 9.2, 3.0 Hz, 1H), 7.42 (s, 2H), 7.09 (d, *J* = 8.8 Hz, 1H), 6.98 (d, *J* = 9.2 Hz, 2H), 4.07 (q, *J* = 6.9 Hz, 2H), 1.38 (t, *J* = 7.0 Hz, 3H), HR-FABMS Calcd for C_15_H_16_N_3_O_4_S (M^+^ + H): 334.0859, Found: 334.0856.

1-(2-Hydroxy-5-nitrophenyl)-3-(*p*-tolyl)thiourea (1c)

Yellow solid (18%), m.p. 133~135 °C, ^1^H-NMR (Acetone-*d*_6_, 400 MHz) *δ* 9.47 (s, 1H), 9.33 (dd, *J* = 15.2, 2.8 Hz, 1H), 8.73 (s, 1H), 7.97 (dd, *J* = 7.6, 3.2 Hz, 1H), 7.43 (s, 2H), 7.25 (d, *J* = 8.4 Hz, 2H), 7.11 (d, *J* = 8.8 Hz, 1H), 2.34 (s, 3H), HR-FABMS Calcd for C_14_H_14_N_3_O_2_S (M^+^ + H): 304.0753, Found: 304.0750.

1-(2-Hydroxy-5-nitrophenyl)-3-(4-isopropylphenyl)thiourea (1d)

Yellow solid (17%), m.p. 160~162 °C, ^1^H-NMR (Acetone-*d*_6_, 400 MHz) *δ* 10.12 (s, 1H), 9.47 (s, 1H), 9.32 (d, *J* = 15.2 Hz, 1H), 8.74 (s, 1H), 7.97 (dd, *J* = 9.2, 3.6 Hz, 1H), 7.48 (s, 2H), 7.32 (d, *J* = 6.8 Hz, 2H), 7.11 (d, *J* = 9.2 Hz, 1H), 2.96–2.92 (m, 1H), 1.25 (d, *J* = 7.2 Hz, 6H), HR-FABMS Calcd for C_16_H_18_N_3_O_3_S (M^+^ + H): 332.1068, Found: 332.1063.

1-(4-Butylphenyl)-3-(2-hydroxy-5-nitrophenyl)thiourea (1e)

Light yellow solid (80%), m.p. 120~122 °C, ^1^H-NMR (Acetone-*d*_6_, 400 MHz) *δ* 10.13 (s, 1H), 9.47 (s, 1H), 7.33 (dd, *J* = 18.4, 3.0 Hz, 1H), 8.73 (s, 1H), 7.97 (dd, *J* = 8.8, 3.8 Hz, 1H), 7.48–7.44 (m, 2H), 7.27 (d, *J* = 8.4 Hz, 2H), 7.10 (d, *J* = 9.2 Hz, 1H), 2.64 (t, *J* = 8.0 Hz, 2H), 1.65–1.57 (m, 2H), 1.39–1.34 (m, 2H), 1.11 (t, *J* = 7.2 Hz, 3H), HR-FABMS Calcd for C_17_H_20_N_3_O_3_S (M^+^ + H): 346.122, Found: 346.1221.

1-(3-Bromophenyl)-3-(2-hydroxy-5-nitrophenyl)thiourea (1f)

Yellow solid (79%), m.p. 213~215 °C, ^1^H-NMR (DMSO-*d*_6_, 400 MHz) *δ* 11.68 (s, 1H), 10.43 (s, 1H), 9.48 (s, 1H), 9.05 (s, 1H), 7.97–7.94 (m, 2H), 7.50–7.33 (m, 3H), 7.00 (d, *J* = 9.6 Hz, 1H), HR-FABMS Calcd for C_13_H_11_BrN_3_O_3_S (M^+^ + H): 367.9699, Found: 367.9697.

### 5.3. Synthesis of 5-Nitro-N-substitutedphenylbenzo[d]oxazol-2-amines (2a–f)

Dried acetonitrile 5 mL was added to potassium hydroxide (KO_2_, 5 eq.) under a N_2_ atmosphere. *N*-(2-hydroxy-5-nitrophenyl)-*N*-substituted-thiourea (100 mg, 1 eq.) solution in acetonitrile was delivered dropwise to the KO_2_-acetonitrile mixture. The reaction mixture was vigorously stirred at room temperature for 16 h under a N_2_ atmosphere. Cold water was added to the reaction mixture, extracted with dichloromethane, followed by washing with brine solution. After drying with anhydrous MgSO_4_ and filtration, the organic solvent was removed under reduced pressure to prepare the compounds 2a–f.

*N*-(4-Methoxyphenyl)-5-nitrobenzo[*d*]oxazol-2-amine (2a)

Yellow solid (42%), m.p. 196~200 °C, ^1^H-NMR (DMSO-*d*_6_, 400 MHz) *δ* 8.28 (d, *J* = 2.4 Hz, 1H), 8.09 (dd, *J* = 8.8, 2.4 Hz, 1H), 7.51 (dd, *J* = 7.6, 2.0 Hz, 2H), 7.38 (d, *J* = 8.8 Hz, 1H), 7.19 (s, 1H), 6.97 (dd, *J* = 6.8, 2.4 Hz, 2H), HR-FABMS Calcd for C_14_H_12_N_3_O_4_ (M^+^ + H): 286.0822, Found: 286.0853.

*N*-(4-Ethoxyphenyl)-5-nitrobenzo[*d*]oxazol-2-amine (2b)

Yellow solid (89%), m.p. 206~209 °C, ^1^H-NMR (DMSO-*d*_6_, 400 MHz) *δ* 9.69 (s, 1H), 8.20 (s, 1H), 8.09 (dd, *J* = 10.2, 2.4 Hz, 1H), 7.75 (d, *J* = 8.8 Hz, 2H), 7.60 (d, *J* = 8.8 Hz, 1H), 6.99 (d, *J* = 9.2 Hz, 2H), 4.07 (q, *J* = 7.1 Hz, 2H), 1.38 (t, *J* = 6.8 Hz, 3H), HR-FABMS Calcd for C_15_H_14_N_3_O_4_ ((M^+^ + H)): 300.0984, Found: 300.0979.

5-Nitro-*N*-(*p*-tolyl)benzo[*d*]oxazol-2-amine (2c)

Light yellow solid (44%), m.p. 219~223 °C, ^1^H-NMR (DMSO-*d*_6_, 400 MHz) *δ* 8.23 (s, 1H), 8.11 (dd, *J* = 8.8, 2.4 Hz, 1H), 7.74 (d, *J* = 6.8 Hz, 2H), 7.61 (d, *J* = 8.4 Hz, 1H), 7.24 (d, *J* = 8.0 Hz, 2H), 3.75 (s, 1H), 2.33 (s, 3H), HR-FABMS Calcd for C_14_H_12_N_3_O_3_ (M^+^ + H): 270.0875, Found: 270.0873.

*N*-(4-Isopropylphenyl)-5-nitrobenzo[*d*]oxazol-2-amine (2d)

Light yellow solid (87%), m.p. 183~185 °C, ^1^H-NMR (DMSO-*d*_6_, 400 MHz) *δ* 9.79 (s, 1H), 8.23 (s, 1H), 8.11 (dd, *J* = 11.2, 2.2 Hz, 1H), 7.77 (d, *J* = 8.8 Hz, 2H), 7.62 (d, *J* = 8.8 Hz, 1H), 7.31 (d, *J* = 7.2 Hz, 2H), 6.75 (s, 1H), 2.97–2.90 (m, 1H), 1.26 (d, *J* = 7,2 Hz, 6H), HR-FABMS Calcd for C_16_H_16_N_3_O_3_ (M^+^ + H): 298.1189, Found: 298.1186.

*N*-(4-Butylphenyl)-5-nitrobenzo[*d*]oxazol-2-amine (2e)

Light yellow solid (84%), m.p. 166~168 °C, ^1^H-NMR (DMSO-*d*_6_, 400 MHz) *δ* 9.79 (s, 1H), 8.23 (s, 1H), 8.11 (dd, *J* = 11.2, 2.4 Hz, 1H), 7.76 (d, *J* = 8.4 Hz, 1H), 7.27 (d, *J* = 8.8 Hz, 2H), 2.63 (t, *J* = 7.6 Hz, 2H), 1.65–1.58 (m, 2H), 1.40–1.35 (m, 2H), 0.94 (t, *J* = 7.4 Hz, 3H), HR-FABMS Calcd for C_17_H_18_N_3_O_3_ (M^+^ + H): 312.1348, Found: 312.1343.

*N*-(4-Bromophenyl)-5-nitrobenzo[*d*]oxazol-2-amine (2f)

Yellow solid (56%), m.p. 229~232 °C, ^1^H-NMR (DMSO-*d*_6_, 400 MHz) *δ* 11.24 (s, 1H), 8.31 (d, *J* = 2.4 Hz, 1H), 8.12–8.08 (m, 2H), 7.76 (d, *J* = 8.8 Hz, 1H), 7.67 (dd, *J* = 8.0, 1.2 Hz, 1H), 7.37 (t, *J* = 8.0 Hz, 1H), 7.27 (d, *J* = 8.0 Hz, 1H), HR-FABMS Calcd for C_13_H_9_BrN_3_O_3_ (M^+^ + H): 333.9822, Found: 333.9824.

### 5.4. Synthesis of N^2^-(Substituted phenyl)benzo[d]oxazole-2,5-diamine Derivatives (3a–f)

A volume of 10 mL of ethanol was added to *N*-(substituted phenyl)-5-nitrobenzo[*d*]oxazol-2-amine (100 mg, 1 eq.) and Tin(II) chloride (12 eq.). The reaction mixture was ultrasonicated for 3 h at room temperature. To the reaction solution was added potassium hydroxide solution and the mixture was extracted with ethyl acetate, followed by washing with brine solution. After drying with anhydrous MgSO_4_ and filtration, the organic solvent was removed under reduced pressure to prepare the compounds 3a–f.

*N*^2^-(4-Methoxyphenyl)benzo[*d*]oxazole-2,5-diamine (3a)

Dark gray solid (33%), m.p. 245~250 °C, ^1^H-NMR (Acetone-*d*_6_, 400 MHz) *δ* 9.07 (s, 1H), 7.74 (d, *J* = 9.2 Hz, 2H), 7.02 (d, *J* = 8.4 Hz, 1H), 6.94 (d, *J* = 8.8 Hz, 2H), 6.73 (d, *J* = 2.4 Hz, 1H), 6.42 (d, *J* = 8.4 Hz, 1H), 4.44 (s, 1H), 3.79 (s, 3H), ^13^C-NMR (Acetone-*d*_6_, 100 MHz) *δ* 159.67, 156.21, 146.54, 145.04, 141.41, 120.11, 115.05, 109.20, 109.15, 103.57, 55.80, HR-FABMS Calcd for C_14_H_14_N_3_O_2_ (M^+^ + H): 256.108, Found: 256.1081.

*N*^2^-(4-Ethoxyphenyl)benzo[*d*]oxazole-2,5-diamine (3b)

Dark gray solid (31%), m.p. 158~160 °C, ^1^H-NMR (Acetone-*d*_6_, 400 MHz) *δ* 9.06 (s, 1H), 7.73 (d, *J* = 9.2 Hz, 2H), 7.02 (d, *J* = 8.4 Hz, 1H), 6.93 (d, *J* = 6.8 Hz, 2H), 6.73 (s, 1H), 6.42 (dd, *J* = 8.8, 2.4 Hz, 1H), 4.41 (s, 2H), 4.03 (q, *J* = 6.9 Hz, 2H), 1.36 (t, *J* = 7.0 Hz, 3H), ^13^C-NMR (Acetone-*d*_6_, 100 MHz) *δ* 159.70, 155.50, 146.52, 145.03, 141.41, 133.37, 120.11, 115.68, 109.21, 109.15, 103.57, 64.29, 15.27, HR-FABMS Calcd for C_15_H_16_N_3_O_2_ (M^+^ + H): 270.1239, Found: 270.1237.

*N*^2^-(*p*-Tolyl)benzo[*d*]oxazole-2,5-diamine (3c)

Light gray solid (37%), m.p. 185~188 °C, ^1^H-NMR (Acetone-*d*_6_ 400 MHz) *δ* 9.16 (s, 1H), 7.71 (d, *J* = 9.2 Hz, 2H), 7.17 (d, *J* = 8.4 Hz, 2H), 7.03 (d, *J* = 8.8 Hz, 1H), 6.75 (s, 1H), 6.44 (dd, *J* = 8.8, 2.2 Hz, 1H), 4.44 (s, 2H), 2.30 (s, 3H), ^13^C-NMR (Acetone-*d*_6_, 100 MHz) *δ* 159.46, 146.55, 144.95, 143.47, 141.35, 137.99, 127.62, 118.70, 109.34, 109.27, 103.64, 34.29, 24.53, HR-FABMS Calcd for C_14_H_14_N_3_O (M^+^ + H): 240.113, Found: 240.1131.

*N*^2^-(4-Isopropylphenyl)benzo[*d*]oxazole-2,5-diamine (3d)

Dark gray solid (32%), m.p. 174~175 °C, ^1^H-NMR (Acetone-*d*_6_, 400 MHz) *δ* 9.17 (s, 1H), 7.74 (d, *J* = 4.4 Hz, 2H), 7.24 (d, *J* = 8.4 Hz, 2H), 7.04 (d, *J* = 8.4 Hz, 1H), 6.75 (s, 1H), 6.44 (dd, *J* = 7.6, 2.4 Hz, 1H), 4.44 (s, 2H), 2.93–2.86 (m, 1H), 1.27 (s, 3H), 1.23 (s, 3H), ^13^C-NMR (Acetone-*d*_6_, 100 MHz) *δ* 157.01, 146.58, 144.96, 137.77, 132.17, 130.26, 118.60, 109.34, 109.27, 103.64, 20.81, HR-FABMS Calcd for C_16_H_18_N_3_O (M^+^ + H): 268.1447, Found: 268.1444.

*N*^2^-(4-Butylphenyl)benzo[*d*]oxazole-2,5-diamine (3e)

Gray solid (55%), m.p. 138~141 °C, ^1^H-NMR (Acetone-*d*_6_, 400 MHz) *δ* 9.17 (s, 1H), 7.73 (d, *J* = 8.8 Hz, 2H), 7.19 (d, *J* = 8.8 Hz, 2H), 7.04 (d, *J* = 8.4 Hz, 1H), 6.75 (s, 1H), 6.44 (dd, *J* = 8.4, 2.4 Hz, 1H), 4.44 (s, 2H), 2.59 (t, *J* = 7.8 Hz, 2H), 1.63–1.56 (m, 2H), 1.41–1.32 (m, 2H), 0.93 (t, *J* = 14.4 Hz, 3H), ^13^C-NMR (Acetone-*d*_6_, 100 MHz) *δ* 159.43, 146.57, 144.97, 141.35, 137.93, 137.38, 129.67, 118.78, 118.61, 109.34, 109.28, 103.65, 35.63, 34.76, 23.00, 14.29, HR-FABMS Calcd for C_17_H_20_N_3_O (M^+^ + H): 282.1604, Found: 282.1601.

*N*^2^-(3-Bromophenyl)benzo[*d*]oxazole-2,5-diamine (3f)

Yellowish brown solid (85%), m.p. 168~170 °C, ^1^H-NMR (Acetone-*d*_6_, 400 MHz) *δ* 9.72 (s, 1H), 7.87 (d, *J* = 7.6 Hz, 2H), 7.51 (d, *J* = 8.4 Hz, 1H), 7.40 (t, *J* = 8.0 Hz, 2H), 7.09 (t, *J* = 7.2 Hz, 2H), ^13^C-NMR (DMSO-*d*_6_, 100 MHz) *δ* 159.37, 146.20, 143.48, 138.27, 127.70, 129.05, 128.11, 122.62, 117.90, 116.10, 111.02, 109.64, HR-FABMS Calcd for C_13_H_11_BrN_3_O (M^+^ + H): 304.008, Found: 304.0079.

### 5.5. Synthesis of N^2^-(4-Ethylphenyl)-N^5^-methylbenzo[d]oxazole-2,5-diamine (4a)

A volume of 10 mL of 1,2-dichloroethane was added to *N*^2^-(4-ethylphenyl)benzo[*d*]oxazole-2,5-diamine (0.39 mmol, 1 eq) in formaldehyde (0.39 mmol, 1 eq.), sodium triacetoxyborohydride (0.55 mmol, 1.4 eq) and triethylamine (0.39 mmol, 1 eq.) and stirred at room temperature for 24 h. To the reaction solution was added saturated sodium carbonate solution, and the mixture was extracted with 30 mL of ethyl acetate, followed by washing with brine solution. After drying with anhydrous MgSO_4_ and filtering under reduced pressure, the organic solvent was removed under reduced pressure. The crude product was purified by column chromatography using *n*-hexane and ethyl acetate as an eluent to obtain the compound 4a.

*N*^2^-(4-Ethylphenyl)-*N*^5^-methylbenzo[*d*]oxazole-2,5-diamine (4a)

Dark yellow solid (24%), m.p. 140~142 °C, ^1^H-NMR (CDCl_3_, 400 MHz) *δ* 7.49 (d, *J* = 8.8 Hz, 2H), 7.21 (d, *J* = 8.8 Hz, 2H), 7.12 (d, *J* = 8.8 Hz, 1H), 6.77 (s, 1H), 6.39 (dd, *J* = 8.8, 2.4 Hz, 1H), 2.86 (s, 3H), 2.64 (q, *J* = 8.0 Hz, 2H), 1.01 (t, *J* = 7.4 Hz, 3H), ^13^C-NMR (Acetone-*d*_6_, 100 MHz) *δ* 159.39, 148.90, 145.07, 140.98, 138.82, 137.96, 129.10, 118.66, 109.39, 107.50, 100.66, 31.37, 28.87, 16.35, HR-FABMS Calcd for C_16_H_18_N_3_O (M^+^ + H): 268.1444, Found: 268.1444.

### 5.6. Synthesis of N-(2-((4-Ethylphenyl)amino)benzo[d]oxazole-5-Yl)acetamide (4b)

A volume of 30 mL of acetic acid was added to *N*^2^-(4-ethylphenyl)benzo[*d*]oxazole-2,5-diamine [15] (0.79 mmol, 1 eq.) in acetic anhydride (1.18 mmol, 1.5 eq.) and stirred at room temperature for 24 h. Organic solvent was removed under reduced pressure to prepare the compound. The compound was recrystallization in *n*-hexane to give the compound 4b.

*N*-(2-((4-Ethylphenyl)amino)benzo[*d*]oxazole-5-yl)acetamide (4b)

Dark gray solid (72%), m.p. > 250 °C, ^1^H-NMR (CDCl_3_, 400 MHz) *δ* 7.56 (s, 1H), 7.47 (d, *J* = 4.8 Hz, 2H), 7.30–7.21 (m, 5H), 7.15 (q, *J* = 7.6 Hz, 2H), 1.24 (t, *J* = 7.6 Hz, 2H), ^13^C-NMR (Acetone-*d*_6_, 100 MHz) *δ* 172.09, 168.69, 159.84, 144.66, 144.32, 139.23, 137.61, 137.20, 129.19, 118.89, 113.99, 113.89, 109.17, 109.10, 28.87, 24.30, 16.34, HR-FABMS Calcd for C_17_H_18_N_3_O_2_ (M^+^ + H): 296.1393, Found 296.1394.

### 5.7. Synthesis of N-(2-((4-Ethylphenyl)amino)benzo[d]oxazole-5-Yl)isobutyramide (4c)

Thirty mL of acetic acid was added to *N*^2^-(4-ethylphenyl)benzo[*d*]oxazole-2,5-diamine (0.39 mmol, 1 eq) in isobutyric anhydride (0.59 mmol, 1.5 eq) and stirred at room temperature for 24 h. After that, organic solvent was removed under reduced pressure to prepare the compound. The crude product was purified by column chromatography using *n*-hexane and ethyl acetate as an eluent to prepare the compound 4c.

*N*-(2-((4-Ethylphenyl)amino)benzo[*d*]oxazole-5-yl)isobutyramide (4c)

Gray solid (28%), m.p. 151~154 °C, ^1^H-NMR (Acetone-*d*_6_, 400 MHz) *δ* 8.96 (s, 1H), 7.95 (s, 1H), 7.76 (d, *J* = 6.4 Hz, 2H), 7.45 (d, *J* = 4.8 Hz, 1H), 7.30 (d, *J* = 8.8 Hz, 1H), 7.23 (d, *J* = 9.2 Hz, 2H), 3.99 (s, 2H), 3.47 (s, 3H), 2.63 (q, *J* = 7.6 Hz, 2H), 1.93 (s, 2H), 1.22 (t, *J* = 7.6 Hz, 3H), ^13^C-NMR (Acetone-*d*_6_, 100 MHz) *δ* 168.42, 159.91, 145.03, 144.33, 139.21, 137.59, 136.01, 129.16, 118.88, 114.58, 109.71, 109.13, 72.94, 59.43, 28.86, 16.34, HR-FABMS Calcd for C_19_H_22_N_3_O_2_ (M^+^ + H): 324.1708, Found: 324.1707.

### 5.8. Synthesis of N-(2-((4-Ethylphenyl)amino)benzo[d]oxazole-5-Yl)-2-methoxyacetamide (4d)

Ten mL of acetic acid was added to *N*^2^-(4-ethylphenyl)benzo[*d*]oxazole-2,5-diamine (0.79 mmol, 1 eq) in 2-methoxyacetyl chloride (1.2 mmol, 1.5 eq.) and stirred at 100 °C for 30 min. The mixture was cooled to room temperature and ice water was added on it. After that, the mixture was filtered under reduced pressure and the organic solvent was removed under reduced pressure to prepare compound 4d.

*N*-(2-((4-Ethylphenyl)amino)benzo[*d*]oxazole-5-yl)-2-methoxyacetamide (4d)

Gray solid (76%), m.p. > 250 °C, ^1^H-NMR (CDCl_3_, 400 MHz) *δ* 8.27 (s, 1H), 7.69 (s, 1H), 7.49 (d, *J* = 8.8 Hz, 2H), 7.35 (dd, *J* = 8.4, 2.2 Hz, 1H), 7.25 (s, 1H), 7.23 (d, *J* = 8.0 Hz, 2H), 4.04 (s, 2H), 3.53 (s, 3H), 2.65 (q, *J* = 7.6 Hz, 2H), 1.24 (t, *J* = 7.4 Hz, 3H), ^13^C-NMR (Acetone-*d*_6_, 100 MHz) *δ* 175.83, 159.84, 144.64, 144.32, 139.22, 137.65, 137.35, 129.18, 118.89, 114.16, 109.33, 109.06, 36.71, 28.87, 20.04, 16.33, HR-FABMS Calcd for C_18_H_20_N_3_O_3_ (M^+^ + H): 326.1499, Found: 326.1499.

### 5.9. Biological Evaluation

#### 5.9.1. IL-6 Reporter Gene Assay

HepG2 cells, a human hepatoma cell line, were grown in DMEM (Dulbecco’s Modified Eagle Medium, Wel-GENE Inc, Daegu, Korea) supplemented with 10% (*v*/*v*) fetal bovine serum, streptomycin (100 U/mL) and penicillin (100 U/mL). Cells were plated onto 96-well plates with 5 × 10^4^ cells/well density and incubated until the confluency was about 80% and stimulated with LPS (Sigma-Aldrich; Merck KGaA) at a concentration of 1 μg/mL. Then, culture medium was changed with 50 μL of serum-free DMEM. The construction of pSTAT3-Luc encoding the stat3 binding site was purchased from Clontech laboratories, Palo Alto, CA. A luciferase reporter construct containing TA minimal promoter with four tandem Stat3-binding sites was introduced into the HepG2 cells using Lipofectamine (Thermo Science, Waltham, CA, USA). For example, 0.1 μg pSTAT3-Luc was suspended with 6 μL of serum-free DMEM and 1 μL of plus reagent added. In a separate tube, 0.3 μL of Lipofectamine reagent was added to 6 μL DMEM. After 15 min at room temperature, the two tubes were mixed, incubated for an additional 15 min and the mixture was added to HepG2 cells. Cells were placed in an incubator for 3 h and transfected cells were changed with new culture medium. After 24 h under incubation condition, cells were starved overnight. The compounds were pretreated to starved cells and incubated for 1 h. Then cells were stimulated with IL-6 (10 ng/mL, BD Pharmingen, San Diego, CA, USA) for 3 h. Luciferase assay was performed using a kit purchased from Promega (Madison, WI, USA) using a luminometer. Optical density of samples was measured on a microplate reader at 540 nm. Data were obtained in triplicates of independent experiments and expressed as average ± SD. Statistical significance was calculated and considered statistically significant at *p* < 0.05.

#### 5.9.2. Measurement of IL-1β Production in RAW 264.7 Macrophage

RAW 264.7 (murine macrophage) cells were seeded in a 96-well plate at 5 × 10^4^ cells/mL. The next day, the medium was replaced with fresh medium. After 1 h under incubation condition, the cells were pretreated with test samples for 1 h before stimulation with 500 ng/mL LPS. The cell culture supernatants were collected after 18 h, and the concentration of IL-1β was determined using a commercial enzyme-linked immunosorbent assay kit (Thermo Science, CA, USA) according to the manufacturer’s protocol. Absorbance was measured on microplate reader at 450 nm and 550 nm and the 550 nm values were subtracted from the 450 nm values to correct for optical imperfections in the microplate. Data were obtained in triplicates of independent experiments and expressed as average ± SD. Statistical significance was calculated and considered statistically significant at *p* < 0.05.

#### 5.9.3. RNA Isolation

The Raw 264.7 cell was lysed with 1 mL TRIzol (Invitrogen, San Diego, CA, USA) using homogenizer on ice, then 200 μL of chloroform was added and vigorously vortexed for 15 sec and rested for 3 min to separate the phenol from lysate, then centrifuged at 12,000× *g*, 4 °C for 15 min. The lysate was separated into three layers. The aqueous-top layer contained RNA and was transferred to another tube. The tube was added 500 μL isopropanol and inverted gently four times and kept at −80 °C for 16 h for precipitation. The frozen mixture in the tube was melted on ice then centrifuged at 12,000× *g*, 4 °C for 10 min. The pellet was washed twice with 70% ethanol in RNase free water and centrifuged at 12,000× *g*, 4 °C for 10 min, then dried. The RNA pellet was resuspended with RNase-free water. RNA purity and concentration were determined using Nano Drop ND-1000 spectrophotometer (Daemyung, Korea).

#### 5.9.4. Reverse Transcription-Polymerase Chain Reaction (RT-PCR) and Quantitative Real Time PCR (qPCR)

Isolated RNA was subjected to RT-PCR to convert single-stranded RNA into stable double-stranded complementary DNA (cDNA). Two μg of RNA diluted with diethyl pyrocarbonate (DEPC) water (final volume 20 μL), then added to cDNA premix (ECODRY, Chandler, AZ, USA) that contained reverse transcriptase, deoxy-nucleotide (dNTP), random hexamer primers, and buffer. The tube was incubated at 42 °C for 60 min (reverse transcription) and then at 70 °C for 10 min (reverse transcriptase inactivation). The resulting cDNA was subjected to qPCR using an ABI 7300 real time PCR system (Applied Biosystems, San Francisco, CA, USA). The reaction had a 20 μL total volume, including 2 μL (40 ng) of cDNA, 10 μL of SYBR Green premix (Bioline, Essex, UK), 0.25 pM of each forward and reverse primers, and autoclaved DW. The primer sequences are listed in Table 3.

#### 5.9.5. Measured Footpad Thickness, Body Weight, and Size of Lymph Node

Female C57BL/6 (H-2b) mice (6–8 weeks old) were obtained from the Korea Research Institute of Bioscience and Biotechnology (Chungbuk, Korea) and housed under specific pathogen-free conditions at 21–24 °C and 40–60% relative humidity under a 12 h light/dark cycle. All animals were acclimatized for at least 1 week prior to the experiments. All experimental procedures were approved by the Chungbuk National University Animal Experimentation Ethics Committee. Inflammation of experimental and control groups was induced by subcutaneous injection (SC) on the right foot with Zymosan A from *Saccharomyces cerevisiae*. Mice were divided into eight groups of six mice per group. Compounds 3a and 3e were administered by intraperitoneal injection (IP) at 3, 10, 30 mg/kg, once a day for 7 days. Zymosan A alone (VH) was administered to the control group, and 1 mg/kg of dexamethasone was administered to the other control group once a day for 7 days. Body weight and footpad thickness were measured on the first, third, fourth, and fifth day. On the seventh day, mice were sacrificed before measuring the size of popliteal nodes (Ethic Committee Name: Chungbuk National University Animal Experimentation Ethics Committee. Approval Code: CBNUA 1124-17-0, Approval Date: 1 August 2017).

## 6. Statistical Analysis

All quantitative data are presented as means ± SD from multiple experiments. Data were analyzed using Student’s *t*-test on Sigma Plot 10.0 software. A *p*-value < 0.05 was considered statistically significant.

## Figures and Tables

**Table 1:** The inhibitory effects of 2,5-diaminobenzoxazole derivatives on IL-6/STAT3 signaling and production of IL-1β from macrophages.

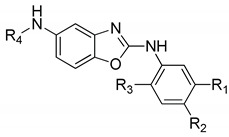						
Compound	R_1_	R_2_	R_3_	R_4_	IL-6/STAT3 Inhibition (%) (10 μg/mL)	IL-1β Production Inhibition (%) (10 μg/mL)
NO LPS					100.0 ± 0.5	100.0 ± 4.1
LPS					0.0 ± 4.9	0.0 ± 7.4
3a	H	OCH_3_	H	H	29.3 ± 4.7 **	92.1 ± 4.5 **
3b	H	OCH_2_CH_3_	H	H	59.1 ± 6.5 **	76.5 ± 2.2 **
3c	H	CH_3_	H	H	17.0 ± 19.7 *	13.7 ± 1.4 *
3d	H	CH(CH_3_)_2_	H	H	65.9 ± 7.2 **	75.3 ± 4.7 **
3e	H	(CH_2_)_3_CH_3_	H	H	71.5 ± 8.2 **	74.4 ± 8.1 **
3f	Br	H	H	H	58.0 ± 5.1 **	79.8 ± 1.4 **
4a	H	CH_2_CH_3_	H	CH_3_	35.9 ± 7.2 **	88.2 ± 6.7 **
4b	H	CH_2_CH_3_	H	COCH_3_	34.4 ± 8.7 **	54.5 ± 4.8 **
4c	H	CH_2_CH_3_	H	COCH(CH_3_)_2_	13.0 ± 0.4 *	88.1 ± 2.2 **
4d	H	CH_2_CH_3_	H	COCH_2_OCH_3_	9.6 ± 2.6 *	26.6 ± 1.0 **
Madindoline					52.0 ± 4.6 **	

* *p* < 0.05, ** *p* < 0.01 vs. LPS inhibition (%). Data are presented as the mean ± SD. Student’s *t*-test was used for comparisons involving two groups (*n* = 3).

**Table 2:** The inhibitory effects of 2,5-diaminobenzoxazole derivatives on IL- 6 and IL-1β mRNA from macrophages.

Raw 264.7	IL-6 mRNA Inhibition (%)		IL-1β mRNA Inhibition (%)	
Compound	1 μg/mL	10 μg/mL	1 μg/mL	10 μg/mL
LPS	0.0 ± 0.91		0.0 ± 0.02	
3a	33.64 ± 1.05 **	44.12 ± 0.25 **	89.01 ± 1.19 **	95.08 ± 0.88 **
3e	65.03 ± 0.52 **	76.10 ± 1.11 **	75.55 ± 2.01 **	78.75 ± 0.98 **
3a + 3e	40.55 ± 0.71 **	45.21 ± 1.03 **	82.55 ± 0.77 **	83.66 ± 0.90 **

** *p* < 0.01 compared with the group treated with the LPS alone. Data are presented as the mean ± SD.

**Table 3:** Sequence of qPCR primers.

Genes	Forward Primer	Reverse Primer
GAPDH	AGAACATCATCCCTGCATCC	GGTCCTCAGTGTAGCCCAAG
IL-1β	TCGTGCTGTCGGACCCATAT	GTCGTTGCTTGGTTCTCCTTGT
IL-6	ACAACCACGGCCTTCCCTACTT	CACGATTTCCCAGAGAACATGTG

## Data Availability

Data are contained within the article.

## Sample Availability

Samples of the compounds are available from the authors.
